# An Ethical Framework for Visitation of Inpatients Receiving Palliative Care in the COVID-19 Context

**DOI:** 10.1007/s11673-022-10173-z

**Published:** 2022-02-17

**Authors:** Bethany Russell, Leeroy William, Michael Chapman

**Affiliations:** 1 Palliative Nexus Research Group, University of Melbourne & St Vincent’s Hospital Melbourne, St Vincent’s Hospital, 41 Victoria Pde, Fitzroy, Victoria 3065 Australia; 2 Department of Palliative Care, St Vincent’s Hospital Melbourne, St Vincent’s Hospital, 41 Victoria Pde, Fitzroy, Victoria 3065 Australia; 3 Supportive & Palliative Care Unit, Eastern Health, Monash University, Melbourne, Australia; 4 Eastern Clinical School, Faculty of Medicine, Nursing & Health Sciences, Monash University, Melbourne, Australia; 5 Public Health Palliative Care Unit, La Trobe University, Wantirna Health, 251 Mountain Highway, Wantirna, 3152 Australia; 6 ANU Medical School, College of Health and Medicine, Australian National University, Canberra, Australia; 7 Department of Palliative Care, Canberra Hospital, PO Box 11, Woden, ACT 2606 Australia

**Keywords:** Palliative Care, Disease outbreaks, COVID-19, Patient-centered care, Family, Bioethics

## Abstract

Human connection is universally important, particularly in the context of serious illness and at the end of life. The presence of close family and friends has many benefits when death is close. Hospital visitation restrictions during the Coronavirus (COVID-19) pandemic therefore warrant careful consideration to ensure equity, proportionality, and the minimization of harm. The Australian and New Zealand Society for Palliative Medicine COVID-19 Special Interest Group utilized the relevant ethical and public health principles, together with the existing disease outbreak literature and evolving COVID-19 knowledge, to generate a practical framework of visiting restrictions for inpatients receiving palliative and end-of-life care. Expert advice from an Infectious Diseases physician ensured relevance to community transmission dynamics. Three graded levels of visitor restrictions for inpatient settings are proposed, defining an appropriate level of minimum access. These depend upon the level of community transmission of COVID-19, the demand on health services, the potential COVID-19 status of the patient and visitors, and the imminence of the patient’s death. This framework represents a cohesive, considered, proportionate, and ethically robust approach to improve equity and consistency for inpatients receiving palliative care during the COVID-19 pandemic and may serve as a template for future disease outbreaks.

## Introduction

Human connection is universally important, particularly in the context of serious illness and at the end of life, yet during the Coronavirus (COVID-19) pandemic various restrictions of social contact were implemented as part of a host of measures to supress virus transmission (Zhou et al. 2020; Tran et al. 2020). In the hospital setting, these restrictions affected all patients—those with a COVID-19 infection and those without. Whilst there are benefits to reducing cross-contamination between patients, families, and staff, and conservation of personal protective equipment (PPE), there are also many negative impacts (Curley, Broden, and Meyer 2020). Hospital visitation restrictions therefore warrant careful consideration to ensure proportionality and the minimization of harm.

As a society, we recognize the importance of access to close family and friends during healthcare experiences. Contact isolation of hospital inpatients colonized by or infected with drug-resistant or contagious pathogens has been associated with psychological harm and poorer patient safety, with up to eight times the usual rate of adverse events (Abad, Fearday, and Safdar 2010). In contrast, family presence during serious illness has been associated with physical and psychological benefits for both patient and family (Goldfarb et al. 2017; Junior et al. 2018). For people receiving palliative care, the presence of family members and loved ones, and the opportunity to say goodbye when death is close, have been described as key components of dying well (the so called “good death”) (Heyland et al. 2006; Meier et al. 2016). Dying alone, however, may result in a “bad death,” a negative experience not just for the dying person but also for family and care staff (Curley, Broden, and Meyer 2020; Nelson-Becker and Victor 2020).

Visitor restrictions instituted in hospitals across the world during COVID-19 thus far have led to significant distress and loss of dignity for people receiving palliative care (Chochinov, Bolton, and Sareen 2020). End-of-life discussions have been compromised and dying alone has become more common (83 per cent during vs 59 per cent pre-pandemic in a Swedish registry study of hospitals and nursing homes), particularly without a relative present (50 per cent vs 17 per cent) due to both visiting restrictions and travel restrictions for dispersed families (Strang et al. 2020). In the hospital setting, already stretched staff are unlikely to be able to fill this gap (the same study noted that the rates of dying with a staff member present did not rise to compensate for the absence of family—49 per cent vs 47 per cent) (Strang et al. 2020). Families may be at risk of disenfranchized grief, prolonged grief disorder, depression, anxiety, or post-traumatic stress disorder if their relative dies alone (Nelson-Becker and Victor 2020; Chochinov, Bolton, and Sareen 2020; Lobb et al. 2010), and the grieving process is further disrupted by concurrent restrictions on after-death rituals and funeral gatherings (Chochinov, Bolton, and Sareen 2020).

Furthermore, visitor restrictions place additional burdens on hospital staff, who have reported feeling “fully responsible” for their patients’ well-being during COVID-19 (Liu et al. 2020). Staff are often tasked with enforcing visitation restrictions in addition to their primary responsibility of providing clinical care (Rogers 2004). Caring for a dying patient who is unable to have loved ones nearby creates moral distress for staff (Wakam et al. 2020; Anderson-Shaw and Zar 2020; Kanaris 2021), of whom junior staff are the most vulnerable (Anderson-Shaw and Zar 2020).

Whilst efforts have been made to embed the use of phone and videoconferencing to facilitate connection (Wang et al. 2020; Ritchey et al. 2020), this cannot adequately substitute for family presence, particularly for seriously unwell patients (Andrist, Clarke, and Harding 2020). This pandemic challenges many fundamentals of contemporary healthcare and palliative care provision (Chapman, Russell, and Philip 2020). Whilst we aspire to “patient-centred” models of care, in times of stress, underlying “hospital-centred” models of care are often revealed (Morano and Calleja-Aguis 2020). It has been suggested that a “paradigm shift” is required to move to “community-centred” models of care that balance competing individual and societal priorities when responding to a pandemic (Nacoti et al. 2020). Noting this broader debate, within this paper we focus on implications for the specific issue of visitation for people who are approaching dying. We argue that for this group of patients, at least *some* visitation should always be permitted, and we present an ethical framework to facilitate this in a transparent, consistent, compassionate, and equitable manner.

## Method

Shortly after the World Health Organization declared COVID-19 a pandemic in March 2020 (World Health Organization 2020), the Australian and New Zealand Society for Palliative Medicine (ANZSPM) convened a COVID-19 Special Interest Group (SIG) with the purpose of providing guidance to healthcare professionals regarding the provision of palliative care in the COVID-19 context. The SIG consisted of thirty-four volunteer members across Australia and New Zealand, all of whom were palliative medicine practitioners, many with specialist qualifications in the field. The SIG met online frequently in multiple subgroups to rapidly progress various areas of work.

The visitation subgroup (BR, LW, MC; all palliative medicine physicians) drafted this guidance with expert advice from an Infectious Diseases physician. The aim was to utilize the relevant ethical and public health principles, amidst the evolving COVID-19 knowledge, to generate a practical framework of visiting restrictions for inpatients receiving palliative and end-of-life care—that is, care focussed on preventing and relieving suffering associated with life-threatening illness (World Health Organization 2002). The ethical considerations in this framework were initially distilled from review of the relevant literature. A process of discussion and deliberation by the authors and the SIG led to determination of several key ethical issues and related principles pertinent to decision-making in this context and described below.

The document underwent extensive peer review by the entire SIG and the Australian COVID-19 Palliative Care Working Group, a multi-disciplinary partnership between ANZSPM, Palliative Care Australia, Palliative Care Nurses Australia, the Australasian Chapter of Palliative Medicine of the Royal Australasian College of Physicians, the End of Life Directions for Aged Care program and Paediatric Palliative Care Australia and New Zealand, CareSearch, and caring@home.

Public health considerations were guided by the Communicable Diseases Network Australia COVID-19 Series of National Guidelines (Communicable Diseases Network Australia 2020). In particular, “suspect” cases described individuals who have met both clinical and epidemiological criteria for COVID-19. The Palliative Care Outcomes Collaboration (PCOC) Phases (Eagar, Green, and Gordon 2004) were used to describe the care plans of patients, specifically, “terminal phase” (death likely in a matter of days) and “deteriorating phase” (worsening existing symptoms or development of new but expected symptoms). “Visitors” included partners, parents, children, siblings, and/or identified next-of-kin. “Adult” was defined as over the age of sixteen. “Essential caregivers” described adult visitors who significantly contributed to the inpatient management of the patient. Their role was defined as providing essential care and support necessary for the patient’s physical, emotional, or social well-being that could not be delivered by the healthcare team or via electronic means, wherein the number and duration of visits should not exceed the time required to provide essential supports only. For example, a familiar caregiver could visit to reassure a patient with cognitive impairment or delirium for the period that they were unsettled.

## Ethical Issues and Principles of Relevance

Decisions to limit visitation to inpatients during the COVID-19 pandemic are informed by numerous ethical concerns and should be acknowledged within the process of determining visiting restrictions. Table 1 groups the ethical principles found in the literature into broader themes but is not exhaustive. There may be additional considerations relevant to decision-making locally. Central to these considerations are several key issues: the formulation of well-being while approaching dying within the pandemic, and the notion of justice within this context. Reconciling these issues is not an easy task and the following discusses these central concerns while indicating how these relate to, and are resolved by, the principles and supporting text within Table 1.

**Table 1:** Ethical principles of relevance

**Ethical Principle(s)**	**Application in the COVID-19 Palliative Care Setting**
Minimize harm and maximize well-being	• Predictable harms related to visitation within the COVID-19 context should be limited, including: - Transmission between healthcare workers, families, and patients. - Patient outcomes with regards to psychosocial and spiritual distress or those impacted by unmet physical needs. - Family caregiver outcomes with regards to psychosocial and spiritual distress and the risk of prolonged grief disorder. - Healthcare worker outcomes with regards to psychosocial and spiritual distress, including moral injury. • Every effort should be made to facilitate digital communication wherever possible (Association for Palliative Medicine of Great • Britain and Ireland 2020).
Equity and respect	• Compassionate, patient-centred care addressing physical, psychological, social, and spiritual needs should be offered to all patients, with or without COVID-19, in all settings as they approach death (Scottish Academy of Medical Royal Colleges 2020; Arya et al. 2020). • Visitation restrictions should be based upon a logical and consistent public health message. Where possible, consistent procedures should be utilized within and between healthcare services. • Providing additional access to visitors for people who are dying supports an equitable approach to care, recognizing heightened needs and a limited timeframe for meaningful connection. • Prioritizing the needs of people who are close to dying through additional support for visitation may create concerns of disadvantage and risk among others which will need to be responded to with clarity, sensitivity, and compassion. Of note, the equitable distribution of PPE for various triaged purposes in times of short supply should be carefully considered.
Honesty and transparency	• There should be transparency regarding the potential for public health needs to be prioritized over the personal autonomy of patients and their caregivers. This should be communicated clearly and compassionately (Rogers 2004). • Visiting restrictions, their rationale and guidance for how to “live with” the rules or make an appeal in special circumstances should be clearly documented and communicated to patients and families (Rogers 2004). This communication should occur when a patient is being transferred from one care setting to another and include how restrictions may change if the patient deteriorates, enters terminal phase or if their COVID-19 status changes (Yardley and Rolph 2020). Clear timeframes should be provided for an expedited appeals process, given the importance of time in end-of-life care. • Visiting restrictions should be included in advance care planning discussions to enable patients and families to make informed decisions regarding ongoing care and preferred place of care or death. Advance care directives made before the COVID-19 pandemic should be reviewed. • The usual practice of notification when a patient rapidly deteriorates, to allow family to be present, should continue.
Flexibility and proportionality	• Where risk and surge levels fluctuate, policies should be reviewed in conjunction with clear guidance at a state and national level (Scottish Academy of Medical Royal Colleges 2020; Arya et al. 2020). For example, in the context of low infection rates, adequate staffing and sufficient personal protective equipment (PPE), it may be feasible to safely facilitate and supervise visitation for all patients. In contrast, during times of high infection rates and high demand on healthcare services it may be necessary to forfeit visitations for most patients. • The potential risks associated with a patient with suspected or confirmed COVID-19 requires proportionate steps to mitigate these risks, relative to patients who are COVID-negative. • The granting of exceptions to communicated rules should be discouraged as it places unreasonable decision-making burden on individual staff members. Furthermore, the ensuing negotiations may damage therapeutic relationships and disrupt health professional teamwork in the delivery of care. • It may be that individual cases do warrant exceptions based on specific circumstances, in recognition of the need for proportionality (Rogers 2004). Here, the risks should be weighed, with focus on the benefits to the patient as the primary focus of care. Ideally, planning should be undertaken in anticipation of needs rather than in a reactive manner. • Support to families, alongside the maintenance of therapeutic relationships and health professional teamwork should be high priorities, underpinned by expert communication skills. In some cases, conversations with individual patients and families about visitation restrictions are best conducted by a staff member not directly involved with the care of the patient, in order to protect those providing clinical care from conflict of interest (Andrist, Clarke, and Harding 2020). Ultimately, the healthcare service should take responsibility for making, communicating, and enforcing the rules and providing accessible support to staff (Rogers 2004).
Capacity and consent	• Patients with decision-making capacity should provide consent to receive each visitor, and where this is not possible, their preference should be sought where possible and respected along with their previously known wishes and the view of a proxy decision maker (Association for Palliative Medicine of Great Britain and Ireland 2020). • Efforts should be made to establish that each visitor understands the risk of exposure to the virus for themselves and their household contacts (Scottish Academy of Medical Royal Colleges 2020).
Community interests and personal autonomy	• A balance must be struck between the best interests of the community, those of individuals, and individual preference to accept personal risks. The need for enforcement of mitigation strategies such as quarantine should be given due importance when weighed against the potential risks for the broader community, including the general local community, the healthcare community (staff and other patients) and the nation.

Consequentialist utilitarian ethics have been highly influential and remain clearly relevant within the ethical deliberations associated with the pandemic (Savulescu, Persson, and Wilkinson 2020). This moral stance weighs choices and provides authority to actions which promote the greatest perceivable net benefit, that is, to act in a way which maximizes benefits and minimizes harms. Within the sphere of visitation restriction this would suggest that limiting or denying visitation is permissible or morally required, as this limits the harms associated with further and preventable spread of COVID-19 to other patients, healthcare workers, and the broader community. Furthermore, limiting the critical resources required to provide healthcare during the constrained time of the pandemic results in fewer deaths and greater well-being for the greater number. The fullest extent of this approach would be to say that complete lockdown of healthcare institutions, with no visitation from people not directly involved in receipt or provision of healthcare, is the most ethical approach to a pandemic.

While acting to achieve a net benefit is a moral strength, determining what constitutes a benefit is problematic. The “good” of saving lives (resulting in an assumed prolongation of well-being for those people and a net benefit for society) needs to be viewed beside other less appreciable and quantifiable benefits and the subtle but important harms that such a focus can engender. A common recent example of these challenges relates to triage of critical resources (such as ventilators) within the pandemic. While approaches to triage decision-making often hinge on the maximizing of some benefits, there is also an acknowledgment that other moral considerations, such as the justice of the decisions and the approach to making them, are highly important (Paton 2020; Jöbges et al. 2020). Acting with justice can be argued as improving the well-being of our community and thus is not inconsistent with a utilitarian approach, however, quantifying and balancing this against other principles is a challenge. Other contexts increase this moral complexity and it has been noted that the more subtle implications for well-being from lockdown rules makes anticipating net benefit highly fraught (Savulescu, Persson, and Wilkinson 2020).

Relating this to visitation for people receiving palliative care, well-being will be influenced by a number of under-appreciated concerns. As we have described, dying in isolation harms the individual approaching dying, their close community, and those providing care (Chapman, Russell, and Philip 2020). Well-supported dying can conversely lead to healing, growth, and joy—subtle but meaningful benefits even in tragic circumstances (Downie 2012). Hospitalized people who are close to dying and their families should be offered choices, within the possibilities available, to inform what occurs and to ensure this is as close as possible to what the patient and family feel is best for them. The personal interests of those involved need to be considered in balance with the outcomes for the community. While the harms of cross-exposure within the hospital contexts would be significant, the risks of this occurring continue to decrease with increasingly sophisticated understanding of COVID-19 and infection control approaches. Within the context of visitation of palliative patients in hospitals, maximizing well-being lies in favour of greater access and availability than has often been the case.

An important additional issue to consider is the issue of justice. Justice, considered as fairness, is often associated with the notion of equality, that all should have access to the same right, benefits, and responsibilities. A claim for equity, however, would suggest that those at a disadvantage should have additional rights and opportunities to diminish their disadvantage. Concerns that COVID-19, and the healthcare and social responses to the pandemic have highlighted, and entrenched injustice have been frequently raised during the pandemic (Fowers et al. 2021). Noting this, we would also suggest that just actions have not been extended to those at risk of dying due to the lack of recognition of concerns relating to their well-being, as discussed above, and due to frequent inequities of their hospital care associated with visitation. Most simply, for people who are close to dying there is less time and more at stake relating to their contact with others, and therefore their needs should be given a greater priority over other hospitalized people who are expected to recover. While a compassionate approach to visitation for dying people has been described as an obtainable “exemption” from usual rules, decision-making relating to this can be slow, ad-hoc, and reliant on those at a disadvantage making a case for just treatment.

Justice, as equity, is therefore likely a beneficial consideration in visitation for people receiving palliative care and close to dying. As mentioned, a moral focus on justice is not inconsistent with utilitarian ethics but may be additionally be clarified through virtue ethics. Virtue ethics suggests that virtues underlie living well and moral behaviour (Zagzebski 1996). Virtues have various characteristics but are often considered sensitive and responsive traits which relate to living well, show up in how we act and can be enhanced through practice and support (Fowers et al. 2021). Acting with virtue therefore requires the possession of a moral disposition and a discriminating awareness and wisdom to enact that virtue through behaviour which is appropriate to the context. Virtues have an established role in healthcare ethics, and virtues, such as being just, can relate to the personal acts of healthcare providers and to institutions. Virtues and the moral behaviours associated with them are also closely related to central elements of palliative care such as the principle of non-abandonment and a focus on holistic care for the person (Sheahan and Brennan 2020).

In the context of decisions around hospital visitation for people with palliative needs within the pandemic, we would suggest that enacting the virtue of justice entails individuals and healthcare institutions duly appreciate the specific and important needs of this population and prioritize these needs within a proportionate balance with other factors. Certain populations of people in hospital, such as those with cognitive impairment or who have illnesses whose palliative phase of care may be identified late and close to dying, are at increased risk of their visitation needs remaining unmet. Additional focus on flexible, timely, and person-centred visitation support for such people is required for a just response to these additional challenges. We would also suggest that this focus implies a need for clearly communicated (honest and transparent), proportionate, and consistent approaches to reconciling these concerns. As above, we would argue that the frequent reliance on ad-hoc appeals for compassionate exemptions undermines just approaches to decision-making through making these instances seem an inappropriately exceptional (rather than expected) aspect of care. This case-by-case approach could also expose healthcare providers, tasked with deciding exemptions, to risk of moral injury due to their perception of a difference between their personal ethics and those of the prevailing institution (Akram 2021).

## Framework for Visitation of Inpatients Receiving Palliative Care

We propose three graded levels of visitor restriction to people receiving palliative care in inpatient settings. This includes patients admitted to dedicated inpatient palliative care units and those admitted to hospital wards who were receiving palliative care from their treating team or a specialist palliative care consultation team or who were otherwise deteriorating and expected to die. The framework represents a proportionate and equitable response, attempting to consider the following:

- level of community transmission
- level of demand on health services
- potential COVID-19 status of the visitors
- COVID-19 status of the patient
- phase of disease of the patient (Eagar, Green, and Gordon 2004)

These factors are integrated within the framework. For example, when the level of demand on health services is low, PPE supply and staff capacity to train and supervise visitors may be sufficient to safely allow a dying patient with confirmed COVID-19 to be visited sequentially by several loved ones. However, when demand on health services is high, the minimum access of one visitor may be all that is feasible. Certain resources, such as the structural design regarding single rooms and private bathrooms; the availability of PPE; the bed capacity of the unit or ward and therefore total potential volume of visitor traffic; and the staff available to support patient care may impact how this advice can be interpreted locally.

This advice also assumes several responsibilities of visitors to health facilities for their presence to be safe and sustainable:

- All visitors must comply with recommended screening, agree and be able to undertake relevant training, perform hand hygiene, wear PPE, and undertake the subsequent isolation requirements relevant to their contact as either close contacts, suspected, or confirmed cases (Scottish Academy of Medical Royal Colleges 2020). The PPE must be of the appropriate size for that visitor, including children. There are no specific implications for a visitor to a person with diagnosed or suspected COVID-19 if appropriate use of PPE has been complied with at all times during the visit.
- Visitors must comply with advice relating to diminishing transmission risk within the hospital. This may include advice to stay in the patient’s room only (including using the patient’s bathroom rather than any communal facilities) and either self-supply food and drink or have catering provided by the healthcare facility to avoid use of the ward kitchen and/or nearby food outlets. They may also be directed to leave the hospital or facility immediately after the visit, avoiding public areas, lobbies, cafes etc. Ideally all visitors would be fully vaccinated where available, but lack of vaccination should not prohibit visitation where the person is willing to comply with the measures above.
- A ward log should be kept of all visitation dates and times, with contact information to facilitate contact tracing if required subsequently.
- It may be necessary to prevent admission to visitors who refuse to comply with restrictions. This will be challenging for staff and visitors alike, but despite best efforts miscommunication may occur. It is therefore important to plan how these encounters will be dealt with. Staff who are screening at entry points will need to be able to deal with the emotions of visitors and have communication skills to diffuse situations. Although we hope the involvement of security personnel is not needed, planning for their availability is important to protect healthcare workers.

Table 2 describes a staged response to the impact of COVID-19 in the community and on health services which forms the basis of the visitation framework. Note that triggers for different response stages can occur from the community or from health services. Table 3 presents a framework for an appropriate **minimum access** to visitors for the inpatient palliative care population in each response stage that balances the factors and concerns described above.

**Table 2:** COVID-19 response stages

**Response Stage**	**Purpose of Response**	**Stage Trigger Points**	
		**Community**	**Local Health Service**
**1**	To ensure baseline COVID-19 risks to patients are limited whilst optimising end of life care.	Minimal to no active COVID-19 cases. Unidentified asymptomatic community cases expected to be negligible.	Minimal to no inpatient care with confirmed COVID-19.
**2**	As per stage 1 PLUS ensure COVID-19 community transmission risks are minimised to patients, visitors, and staff.	Multiple active locally acquired COVID-19 cases in contacts of confirmed cases. Minimal to no active locally acquired COVID-19 cases where source is not identified. Unidentified asymptomatic community cases expected to be negligible.	Regular care of confirmed COVID-19 cases in high risk wards.
**3**	Ensure all potential risk can be mitigated to all patients, visitors and staff.	Multiple active locally acquired COVID-19 cases where source is not identified. Unidentified asymptomatic community cases unable to be quantified**.**	High volumes of confirmed COVID-19 cases.

**Table 3:** Framework for visitation of inpatients receiving palliative care

**Stage 1**	
**Patients with no suspected COVID-19** Physical distancing must be maintained	**Patients with suspected/confirmed COVID-19** Physical distancing must be maintained PPE and masks must be worn
For patients NOT in deteriorating or terminal phase • Limit of 2 visitors at any one time from a list of 4 nominated visitors drawn up on admission, in discussion with the patient and/or caregiver • Any changes to the nominated visitors should be made on a case-by-case basis • Maintain usual visiting hours as much as possible • Visits should permit a minimum of 2 hours per day • Family members who are under the age of 16 may visit* • Religious, spiritual, or community leaders who are not employed by the health service are permitted to visit but must be included in the maximum of 2 visitors at any one time, although not the list of 4 nominated visitors • “Essential caregivers” may visit in addition to the above, including overnight, by negotiation as required and according to the patient’s care plan, but must be included in the maximum of 2 visitors at any one time, although not the list of 4 nominated visitors	For patients NOT in deteriorating or terminal phase • One visitor per day from a list of 4 nominated visitors drawn up on admission, in discussion with the patient and/or caregiver • Any changes to the nominated visitors should be made on a case-by-case basis • Maintain usual visiting hours as much as possible • Visits are for a maximum of 2 hours per day • Family members who are under the age of 16 may visit* and at these times two visitors are permitted together (the child and the designated adult visitor) to allow for supervision and support • An “essential caregiver” may visit in addition to the above, including overnight, by negotiation as required and according to the patient’s care plan • Every effort should be made to facilitate digital communication wherever possible
For patients in deteriorating or terminal phase Visiting restrictions should be lifted and revert back to usual local practice in terms of numbers and duration of visit. If this is not deemed acceptable due to practical management of visitors in the clinical space, or concerns regarding confusion of public health social distancing directives, then a minimum of 2 visitors at any one time would be required.	For patients in deteriorating or terminal phase As above
**Stage 2**	
**Patients with no suspected COVID-19** Physical distancing must be maintained	**Patients with suspected/confirmed COVID-19** Physical distancing must be maintained PPE and masks must be worn
For patients NOT in deteriorating or terminal phase • Limit of 2 visitors at any one time, ideally the same two people throughout the admission • Visits are for a maximum 2 hours per day • No visitors under the age of 16 • Religious, spiritual, or community leaders who are not employed by the health service may not be permitted to visit, and so additional planning to meet spiritual needs may be required • “Essential caregivers” may visit in addition to the above, including overnight, by negotiation as required and according to the patient’s care plan but must be included in the maximum of 2 visitors at any one time	For patients NOT in deteriorating or terminal phase • No visitors allowed, except an “essential caregiver,” including overnight, by negotiation as required and according to the patient’s care plan • Every effort should be made to facilitate digital communication wherever possible
For patients in deteriorating or terminal phase As above, and additionally: • The limit of 2 visitors at any one time will be maintained during terminal and bereavement phase • One adult visitor is permitted to sleepover, subject to local protocol but must be included in the maximum of 2 visitors at any one time • Family members who are under the age of 16 may visit*	For patients in deteriorating or terminal phase As above, and additionally: • One adult visitor, ideally the same person throughout the admission • Visits are for a maximum of 2 hours per day • Family members who are under the age of 16 may visit* and at these times two visitors are permitted together (the child and the designated adult visitor) to allow for supervision and support
**Stage 3**	
**Patients with no suspected COVID-19** Physical distancing must be maintained Masks must be worn	**Patients with suspected/confirmed COVID-19** Physical distancing must be maintained PPE and masks must be worn
For patients NOT in deteriorating or terminal phase • No visitors allowed, except an “essential caregiver,” including overnight, by negotiation as required and according to the patient’s care plan • Every effort should be made to facilitate digital communication wherever possible	
For patients in deteriorating or terminal phase • One adult visitor, the same person throughout the admission • Visits are for a maximum of 2 hours per day • Every effort should be made to facilitate digital communication wherever possible	

*Where visitors under the age of 16 are permitted to visit, they must:

• Be able to comply with handwashing, not touching the environment, and wearing any required PPE or are babies that can be carried throughout the visit

• Be accompanied by an adult at all times who is responsible to supervise them and ensure compliance with infection control measures

• Limit visits to a maximum of 2 hours, although shorter times may be appropriate depending on their ability to maintain compliance with infection control measures

• Be counted as one of the list of nominated visitors

## Visitation by Interstate or Overseas Visitors

Where a visitor wishes to travel from interstate or overseas to visit a person in an inpatient palliative care setting, this should be facilitated wherever possible, depending on the level of community transmission and staff capacity to supervise visitors. Where a period of quarantine is usually required, requests for written permissions to break quarantine for the purpose of visiting the health service should be responded to by the local health service and subsequently the governmental health department in a timely and transparent manner. If granted, the terms of the permission should stipulate that the visitor should wear a surgical mask at all times, including when travelling to and from the hospital or facility and observe strict hand hygiene throughout their visit. The visit must be cancelled if the visitor develops symptoms consistent with COVID-19 or tests positive on screening. Consideration should also be given to the visitor’s needs during potential bereavement and they should be informed of the restrictions that will apply, particularly any requirement to complete the remaining quarantine period without physical contact with other mourners.

Ideally, a stratified public health response for the granting of compassionate travel exemptions should be agreed upon nationally and internationally, clearly communicated to the general public and applied in a consistent, transparent manner.

## Discussion

To the best of our knowledge, this is the first presentation of a scientifically based, specific, graded and ethically informed framework for visitation of inpatients receiving palliative care during the COVID-19 pandemic. Our review found that formal visitation policies are either not available, or if available, are not published and defended. The novel framework provides practical guidance that can be applied in a variety of inpatient settings and embeds the ethical principles articulated.

The implementation of visitor restrictions places an additional burden on the interdisciplinary team. Restrictions therefore need to be practical for successful implementation. The screening and tracking of visitors and any exemptions permitted for each patient should be facilitated at an organization systems level and be carefully organized to minimize disruption to the workloads of clinical and clerical staff. Practical guidance is available on instruction, supervision and monitoring of PPE use and physical distancing during visits (Australian College of Critical Care Nurses and Australasian College for Infection Prevention and Control 2020) but will require dedicated nursing time.

It is important to consider public expectations, which may be dynamic, during the COVID-19 pandemic. Messaging to society from governments, experts, healthcare providers, and the media can considerably influence community expectations and hence the acceptability of visiting limitations. Guidelines should therefore be transparent and based upon logical arguments from current evidence, with a consistent communications strategy from leaders across all sectors of the community.

Due to the uncertainties of prognostication and the potential for disease reversibility clinical care and communication may be improved through use of timeframes to review treatment and visiting restrictions. At the end of the agreed timeframe, care can be reviewed and changes to visiting arrangements discussed within a family meeting. Sub-dividing an unknown timeframe into manageable periods through pre-agreement of expectations and follow-up, can make uncertainty more tolerable for patients and families.

The cultural aspects of end-of-life care should always be respected. This obligation does not change within a pandemic. Reconciling some cultural values and public health expectations during the COVID-19 pandemic may be challenging. Many cultures assume visitation from large groups and families during illness. It may be that a key spokesperson (who may or may not be a relative of the patient) can support the discussion of needs with healthcare providers. Such challenges need to be explored with sensitivity, seeking a solution in partnership. Explanations of the visiting policy should be clear and accompanied by the reasoning according to the knowledge about the SARS-CoV-2 virus.

COVID-19 has had a global impact. We acknowledge that the approach to addressing the issue of visitation for people who are imminently dying and the subsequent recommendations outlined within this paper, are influenced by socio-economic factors, the approach to healthcare and cultural facets within our region (Australia and New Zealand). Our specific recommendations may therefore not align with other contexts, potentially limiting their utility. Furthermore, this guidance should be understood as describing an acceptable **minimum level** of visitation. These recommendations should be revised and adapted in response to new evidence and when population vaccination is achieved. Future revisions should be referenced with broader groups to ensure intersectional relevance. However, we argue that the ethical framework that has been outlined and our recommendations are of practical value to others during this pandemic. Much of our approach could and should be adopted widely as a matter of urgency, since there is a moral imperative for further attention and action regarding these matters to minimize harm as the COVID-19 pandemic continues to unfold. This work could perhaps also act as a guide in negotiating responses to similar issues in future times of risk and compromise.

## Conclusion

Achieving the right balance between compassionate care and public health imperatives is a moral necessity that will require expert communication, flexibility, creativity, and genuine understanding of what is important to our community. This will best be achieved through carefully balancing these objectives with compromise, informed by ongoing assessments of the risks and benefits of acting otherwise. Consumer input from varied stakeholder groups would be valuable to ensure further policy development regarding visiting restrictions is as culturally appropriate as possible, and ideally, to enable international consensus to be reached (Yardley and Rolph 2020). Further work would be worthwhile to evaluate the impact of any policies already in place at healthcare institutions and co-develop new approaches to meet institutional need. In the meantime, this framework represents a cohesive, considered, and ethically robust approach that is urgently needed to improve equity and consistency for people receiving palliative care during the COVID-19 pandemic and may have additional value as a template for future epidemics and pandemics.
